# The Effect of Omega-3 Fatty Acids in Patients With Active Rheumatoid Arthritis Receiving DMARDs Therapy: Double-Blind Randomized Controlled Trial

**DOI:** 10.5539/gjhs.v8n7p18

**Published:** 2015-11-03

**Authors:** Elham Rajaei, Karim Mowla, Ali Ghorbani, Sara Bahadoram, Mohammad Bahadoram, Mehrdad Dargahi-Malamir

**Affiliations:** 1 Department of Rheumatology, Golestan Hospital, Ahvaz Jundishapur University of Medical Sciences, Ahvaz, Iran; 2 Department of Nephrology, Golestan Hospital, Ahvaz Jundishapur University of Medical Sciences, Ahvaz, Iran; 3 Medical Student Research Committee & Social Determinant of Health Research center, Ahvaz Jundishapur University of Medical Sciences, Ahvaz, Iran

**Keywords:** rheumatoid arthritis, Omega-3 Fatty Acids, disease activity score, DMARDs, analgesic

## Abstract

**Background:**

Rheumatoid arthritis is a symmetric peripheral polyarthritis of unknown etiology that, untreated or if unresponsive the therapy, typically leads to deformity and destruction of joints due to erosion of cartilage and bone. Omega-3 fatty acids have been shown to reduce morning stiffness, the number of tender joints and swollen joints in patients with rheumatoid arthritis. This study is designed for evaluation of omega-3 effects on disease activity and remission of rheumatoid arthritis in DMARDs treated patients and on weight changes and reduction of analgesic drugs consumption versus placebo.

**Methods:**

Sixty patients with active rheumatoid arthritis (49 female and 11 male) underwent rheumatologist examination and disease activity score were calculated. Then patients were enrolled in this 12 week, double blind, randomized, placebo- controlled study. The patients in both groups continued their pre study standard treatment. The patients were visited every 4 weeks, 4 times and data were recorded.

**Results:**

Significant improvement in the patient’s global evaluation and in the physician’s assessment of disease was observed in those taking omega-3. The proportions of patients who improved and of those who were able to reduce their concomitant analgesic medication were significantly greater with omega-3 consumption. There were no weight changes.

**Conclusion:**

Daily supplementation with omega-3 results has significant clinical benefit and may reduce the need for concomitant analgesic consumption without weight changes.

## 1. Introduction

Rheumatoid arthritis (RA) is a chronic inflammatory disease of the joints. Despite a high prevalence (1%) and widespread debility in most patients, it is currently cureless and most treatments only affect the progression or symptoms of the disease.

The effect of omega-3 fatty acids in the treatment of inflammatory diseases, including rheumatoid arthritis has been shown (Lee et al., 2012). Increased omega - 3 fatty acid intake leads to a reduce in arachidonic acid binding to cell membranes, causing a marked inflammatory response and stronger inflammatory markers such as tumor necrosis factor notably reduce. Moreover, the diet’s omega-3 leads to reduced production of pro-inflammatory cytokines (PGE2, LTB4) and cartilage-degrading enzymes by increasing n-3 FA and reducing arachidonic acid (Cleland et al., 2006). Also, some studies relate the relative lack of omega-6 fatty acids, which are eventually metabolized into arachidonic acid and inflammatory eicosanoids, to the effectiveness of PU-FAS (Bhangle & Kolasinski, 2011). Furthermore, it is believed that, despite low levels of omega 3, traditional dairy products can have anti-inflammatory effects in the same way by effecting conjugated linoleic acids (Aryaeian et al., 2014; Skulas-Ray, 2015). Although several studies have been conducted in relation to the effect of omega-3 in patients with rheumatoid arthritis, due to the small sample size and low statistical power of previous studies, there is still disagreement on this issue (Berbert et al., 2005; Lee et al., 2012; Aryaeian et al., 2014; Calder, 2015; Hershman et al., 2015; Klein & Gay, 2015; Skulas-Ray, 2015). However, most of these studies have been conducted on patients with long-term illness (Long standing RA) and until now, no studies have been done on the long-term prognosis of newly diagnosed (Early RA) patients with active rheumatoid arthritis (RA) with the early use of omega-3 and rheumatic modulators (DMARDs). Therefore, this study aimed to assess the efficacy of omega-3 in standard treatment (DMARDs) in patients with newly diagnosed rheumatoid arthritis to improve clinical characteristics and laboratory findings. Furthermore, considering that recent studies have shown the effect of genetics and environmental factors such as weather on the prevalence and severity of clinical signs and symptoms of rheumatoid arthritis (Calder, 2015), this study was carried out in the warm climate of this area.

## 2. Methods

### 2.1 Study Population

This randomized double blind clinical trial was performed on 60 patients (49 females, 11 males) who attended the Rheumatology Clinic of Ahvaz Golestan Hospital with active RA (according to the Association of Rheumatology America (ACR)), over a 12 week period (Arnett et al., 1988). An inclusion criterion was patient selection by two rheumatologists after clinical evaluation and diagnosis of their illness. Exclusion criteria included a diagnosis of over 6 months, bone deformities, severe concomitant diseases such as metabolic and gastrointestinal diseases, the functional group IV criteria for ACR, medication dose fluctuation during the study, use of Omega-3 fatty acids supplements, digestive intolerance and severe infection, AST, ALT, or Creatinine levels higher than 1.5 times the maximum normal limit, total bilirubin levels more than 1.8 mg/dL, incompletely finishing the 12 week treatment course, discontinuation of therapy due to side effects, or absence from periodical examination sessions (absence of more than one session). After thorough explanation of the purposes of this study, a written consent was obtained from patients with tendency to participate. After reviewing the study protocol and confirmation by the Ethics Committee of Ahvaz University of Medical Sciences the study began.

### 2.2 Study Intervention

Eligible subjects were allocated randomly within blocks after selection based on criteria such as age, sex, drug consumption and duration of disease. No significant differences were seen between the groups at baseline. Also, confounding factors such as the number of drugs were controlled in the 2 groups, and significant differences were not found between them. Patients were randomized placed into one of two (omega-3 and placebo) groups. A person other than the researcher encoded the packages containing omega-3 fatty acids (A, B). Packages containing supplements were distributed based on coding and data analysis was done by another person.

Throughout the study period, the standard therapy, including 5 mg prednisone twice a day, 200 mg hydroxychloroquine daily, and 0.2 mg/kg MTX per week were continued in both groups. In addition, all patients in both groups used the same non-steroidal anti-inflammatory drug (NSAID) (25 mg indomethacin three times daily).

The dose of omega-3 and placebo was constant throughout treatment. Patients were instructed to continue their usual diet and physical activity during the study, and refrain from drug dose fluctuations without notice. Placebo drugs which consisted of starch and resembled pharmaceutical omega-3 were developed by Anzan Pharmaceutical Company.

Patients consumed 2 omega - 3 capsules daily which contained 1.8 and 2.1 grams of EPA and DHA, respectively. Afterwards, patients were evaluated every four weeks for three months in terms of clinical and laboratory findings, including ESR, CRP, daily analgesic consumption, number of tender and swollen joints, duration of morning stiffness of the joints in minutes, the physician’s overall assessment of disease activity, patients’ severity of joint pain and the patients’ disease activity using a visual analog scale (VAS). Classification of functional status was calculated according to ACR (Arnett et al., 1988), and disease severity index score (DAS-28) which uses variables for the number of swollen and painful joints, ESR and overall assessment of the patient’s general health status (Landewe et al., 2006). In addition, the ACR-20 and ACR-50 indicators were measured. ACR-20 and ACR-50 represent a 20% and 50% improvement respectively in the number of swollen and tender joints, and at least 3 of 5 other ACR criteria compared to the baseline (Felson, Anderson, Boers et al., 1995; Felson Anderson, Lange et al., 1998).

### 2.3 Statistical Analysis

Data were analyzed by SPSS 20. To describe the subjects’ features, descriptive analysis techniques, such as mean and standard deviation for quantitative variables, and frequency for qualitative variables, were used. To compare the age and number of drugs, ANOVA and to compare gender, Fisher’s exact test was used. To insure normal distribution of mean variables, Kolmogorov- Smiranov Test and Leven’s Test were used respectively to achieve a normal distribution of quantitative variables and equality of variance. ANOVA was used to compare the changes between the two groups. Tukey test was used for comparisons of groups where the difference was statistically significant. The significance level was 0.05 in this study.

## 3. Results

Of the 60 patients who entered the study with a mean age of 42.4 ± 7 years, 49 successfully completed the study (Table 1). In the Omega – 3 group, 5 patients were excluded (2 for refrain from drug use, 1 due to absence, 2 due to drug side effects (1 vomiting, 1 flatulence)). In the group taking placebo 6 patients were excluded; 1 for refrain from drug use, 1 due to absence, 2 due to drug side effects and 2 due to activation of the disease. It should be noted that no major differences were seen between the patients who completed the study and those who were excluded.

**Table 1:** Primary features by gender at the beginning of the study

	Male	Female
Number	11	49
Mean Age	44	42
Age Range	20-61	21-70
RF Rate	100% positive	100% positive
ESR Level	36	38
Morning Stiffness (Minutes)	128	136
Mean Number Of Tender Joints	21	24
Mean Number Of Sensitive Joints	7	10

The control group consisted of 24 women and 6 men and the omega – 3 group consisted of 25 females and 5 males. At the end of the study, mean morning stiffness decreased in the omega - 3 group from 128 minutes to 40 minutes; the average number of tender joints was reduced from 21 to 5 joints; the number of swollen joints dropped from 10 to 3; the average ESR decreased from 39 to 16; and the overall assessment of the disease by the patient and the doctor showed a notable reduction of pain. Changes in body weight in the case group showed no significant difference at the end of the study compared to the beginning. The use of analgesics decreased in the omega-3 group from 25 patients at baseline to 7 at the end of the study (72%). Of these 18 patients, 8 patients (32%) completely discontinued the use of pain medication, and 10 patients (40%) reduced the dose of analgesics, however a statistically significant change was not seen among placebo consumers in reducing the amount of pain medications.

According to T-Test, results showed the drugs effect on morning stiffness, and the number of painful and swollen joints was more than placebo. In addition, in the patient and physician’s judgment, pain in patients using omega - 3 declined. Omega - 3 reduced the ESR in patients and the evaluation of patients, based on DAS 28 criteria, showed that Omega - 3 had positive effects, while no statistically significant changes were seen in the weight in patients in the omega-3 group at the beginning and end of the study (Table 2).

**Table 2:** Comparison of DAS 28 activity based on the number of patients in each group at the beginning and end of the study

	Placebo		Omega-3	
	Baseline	End of the study	Baseline	End of the study
Mild Activity(≤ 3.2)	0	0	0	20
Moderate Activity (>3.2& ≤5.1)	22	24	21	5
Severe Activity(>5.1)	2	0	4	0

**Figure 1:**
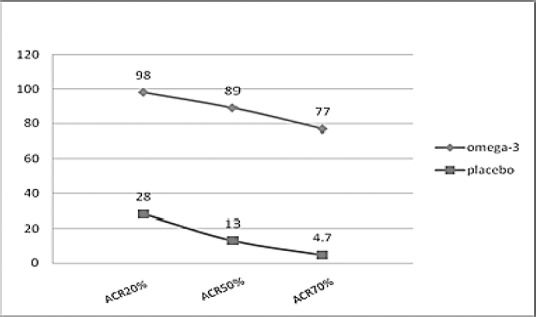
Comparison of improvement in both groups based on ACR scale

50% improvement was seen in tender joints in 89% of patients and 70% improvement in tender joints was achieved in 77% of patients receiving omega-3, while 50% improvement was only seen in 13% of the placebo group. Also, 70% improvement was only seen in 4.7% of patients in the placebo group (Figure 1).

At the end, a survey was conducted on the study. Patients were allowed to answer questions as “good, bad or no effect”. In the group receiving omega - 3, 19 patients (76%) choose good, 2 patients (8%) answered bad and 4 patients (16%) chose the answer no effect. Of the 24 patients receiving placebo, 9 patients (37.5%) chose good, 3 patients (12.5%) bad and 12 (50%) chose the answer no effect.

At the end of the study significant improvement in clinical measures of disease activity, including the number of tender joints, number of swollen joints, physician’s global assessment, patient’s assessment of pain, patient global assessment of the general situation and the severity of the disease in the group of omega-3 post 12 weeks of supplementation were observed (P < 0.05) (Table 3).

**Table 3:** Comparison of omega - 3 and placebo in patients with rheumatoid arthritis at the beginning and end of the study

	Placebo	Omega-3
	Morning Stiffness	
Baseline	**116**	**128**
End of study	**94**	**40**

	Number of tender joints	
Baseline	**24**	**21**
End of study	**20**	**5**

	Number of swollen joints	
Baseline	**7**	**10**
End of study	**5**	**3**

	ESR	
Baseline	**35**	**39**
End of study	**33**	**16**

	CRP	
Baseline	**2**	**2**
End of study	**+2** to **+3**	**0** to **+1**

	RF	
Baseline	**100 +**%	**100 +**%
End of study	**85 +**%	**40 +**%

	Patient’s pain assessment	
Baseline	**8**	**9**
End of study	**8**	**4**

	Doctor’s pain assessment	
Baseline	**4**	**4**
End of study	**5**	**2**

	Weight	
Baseline	**70**	**64.3**
End of study	**71**	**64.5**

	Use of analgesics	
Baseline	**24**	**25**
End of study	**22**	**7**

## 4. Discussion

The treatment effect of omega-3 supplements in conjunction with the standard treatment (DMARDs) in patients with newly diagnosed rheumatoid arthritis on clinical and laboratory findings were studied over 3 months in two groups of patients. The groups were treated with either omega-3 fatty acids in addition to standard medical therapy (DMARDs) or only standard therapy (DMARDs) along with a placebo drug. Comparison was made according to ACR and DAS 28 criteria, and the results showed improvement in many clinical and laboratory characteristics of patients with active rheumatoid arthritis who received diet supplements of omega - 3 along with the standard treatment (DMARDs). In our study, a significant improvement was seen at the end of the twelfth week in 7 clinical variables, morning stiffness of the joints, overall assessment of the patient’s general condition, severity of pain, the physician’s assessment of the patient’s condition, the number of swollen joints, number of tender joints and physical function. In a study by Berbert and his colleagues with a similar dose of Omega-3 PUFAs (3.0 gr/day; 1.8 gr EPA and 1.2 gr DHA) over a 24 week study improvement was seen in pain, morning stiffness and patient global assessment (Berbert et al., 2005). However, some Meta-analyzes have shown that the consumption of omega-3 PUFAs in patients with rheumatoid arthritis had no effect on the inflammation of joints and overall assessment of the patients (Calder, 2015). In a meta-analysis by Lee et al. a significant relationship was not observed between the use of omega-3 and clinical variables (Lee et al., 2012).

Inappropriate placebo pill ingredients such as olive oil, corn oil and soybean oil are used (Berbert et al., 2005; Calder, 2015; Klein & Gay, 2015) with the impression that mono-unsaturated fatty acids are neutral fatty acids (Lee & Park, 2013) whereas, in some studies, the use of olive oil has shown an even greater improvement in disease activity compared to omega-3 (Calder, 2015). Therefore, olive oil cannot be considered as a neutral placebo. In relation to corn and soybean oil immunological effects and improvement in pro-inflammatory conditions have been seen (Miles & Calder, 2012; Klein & Gay, 2015).

Other factors that affect the strength of omega-3 in previous studies can be the background of non-steroidal anti-inflammatory drug use in patients with rheumatoid arthritis. The use of such drugs diverts arachidonic acid substrate from cyclooxygenase pathways to lipoxygenase pathways and as a result, reduces the effect of fish oil on lipoxygenase pathway products (Calder, 2015). In our study, all participants only received indomethacin.

In most studies, linoleic acid intake was not controlled because the omega-6 fatty acid ultimately metabolized into arachidonic acid and inflammatory eicosanoids. Arachidonic acid is an important factor in the production of pro-inflammatory cytokines (Calder, 2015). The diet’s Omega-3 reduces the production of PGE2, LB4 and cartilage degrading enzymes by increasing n-3 FA (Wardhana et al., 2011). Some studies have also shown that omega-3 can lead to the relative lack of omega-6 by competitive inhibition (Bhangle & Kolasinski, 2011). Other studies have mentioned that restricting arachidonic acid intake is also a prerequisite for the anti-inflammatory effects and benefits of omega-3 in patients with rheumatoid arthritis (Miles & Calder, 2012). It is recommend that researchers consider not measuring plasma lipids and patients’ compliance in future studies for a more detailed assessment.

In the final evaluation, 76% of patients receiving omega - 3 expressed satisfaction from participation in this project which was considerably higher than the placebo group (37.5%). While in the placebo group 2 participants were excluded from the study due to worsening of disease activity. In this study the effects of the drug, clinical symptoms, and laboratory findings were reviewed. CRP is mostly used to follow disease activity during the acute phase in patients with rheumatoid arthritis. Multiple studies have shown that consumption of Omega-3 has a special role in reducing inflammatory markers (Calder 2015). In our study the effects of omega - 3, on CRP as a major inflammatory marker, were analyzed, and the results indicate a significant reduction in CRP levels after treatment with omega - 3. These results have been confirmed by other studies (Berbert et al., 2005). Considering the relationship between CRP and the degree of bone destruction and disease activity, the beneficial effects of omega - 3 in combination with DMARDS can be further shown. On the other hand, the effect of omega-3 on CRP in some studies was not clear, and in spite of a 12 week regimen with doses of 1.5, 2 and 6.6 gr/day of omega-3 fatty acids, CRP levels were not significantly different from those in the placebo group (Fenton et al., 2013). It seems as though contradictory results of different studies may be due to different doses of omega-3 fatty acids and the participation of healthy volunteers.

In our study, ESR levels were significantly lower in the omega-3 group compared to the control group, which was also seen in other studies (Olendzki et al., 2011; Klein & Gay, 2015). Olendzki and colleagues, in a study of patients with rheumatoid arthritis, showed a slight (modest) but significant decline in ESR and CRP compared to the baseline values. These remained significant for 18 months for ESR levels but only remained significant for 9 months for CRP levels (Olendzki et al., 2011). On the other hand, due to the possibility of increase from other causes, some meta-analysis studies did not see ESR as an appropriative test (Olendzki et al., 2011). In addition the consumption of omega-3 PUFAs was considered to be ineffective on ESR levels in patients with rheumatoid arthritis. (Felson et al., 1995; Calder, 2015) However, it seems that these results may be due to lack of consideration of the role of high oxidation in patients with rheumatoid arthritis in these studies (Olendzki et al., 2011; Calder, 2015). Some recent studies have shown that the omega-3 fatty acids combined with a low dose of vitamin E, can also reduce the production of inflammatory markers in the blood mononuclear cells leading to a reduction in these patients’ lipid peroxidation, and ultimately reduce the drug’s side effects, particularly heart problems resulting from prolonged use of drugs which is the leading cause of death in patients with rheumatoid arthritis (Zhu et al., 2014). In addition to inflammatory mediators and prothrombotic factors in patients with rheumatoid arthritis, drugs also disrupt the vascular endothelial and influence the development of cardiovascular disease. In our study patients, a reduction in the need to use non-steroidal anti-inflammatory drugs (NSAID) (indomethacin) during 12 weeks of treatment with omega - 3 was observed. These results are consistent with some previous studies (Lee et al., 2012; Klein & Gay, 2015). It seems that consumption of omega-3 in combination with standard drugs (DMARDs) can improve symptoms of cardiovascular problems resulting from the chronic use of such drugs (DMARDs), especially methotrexate (MTX). However, so far little evidence exists to show the impact that control of chronic inflammation has on the reduction of the risk of cardiovascular disease (Tanasescu et al., 2009). No statistically significant differences were seen in the weight in patients between the two groups.

Considering the results of this study and other studies mentioned previously, it appears that in hot climates, use of omega - 3supplements along with DMARDS treatment in patients with active RA can be effective in reducing symptoms such as pain, the need for analgesics, the number of swollen joints, and inflammatory markers that play a large role in joint destruction, and lead to an increase in physical strength. Given that the country’s vast geographic areas are different in terms of climate and weather we recommended that this be evaluated in other areas as well.

## 5. Conclusion

The results of this study show that early use of omega - 3supplements along with DMARDS treatment in patients with newly diagnosed RA can be effective in reducing symptoms.
